# Right place, right time: movement of a fungal effector between subcellular compartments activates plant immune responses

**DOI:** 10.1093/plphys/kiag314

**Published:** 2026-06-01

**Authors:** Josephine H R Maidment

**Affiliations:** Assistant Features Editor, Plant Physiology, American Society of Plant Biologists, MD 20855, United States; PHIM Plant Health Institute, INRAE, CIRAD, Institut Agro, IRD, Univ Montpellier, Montpellier 34980, France; Centre de Biologie Structurale, INSERM, CNRS, Université de Montpellier, Montpellier 34090, France

To successfully colonize a host, pathogens secrete molecules known as effectors into plants. While some effectors remain in the apoplast, others are translocated into the host cell and can subsequently be directed to specific subcellular compartments. Effectors are functionally diverse, but collectively they act to manipulate host physiology and metabolism to promote pathogen virulence. Understanding the molecular functions of effectors can illuminate plant processes targeted by pathogens during infection.

The obligate biotrophic fungus *Erysiphe quercicola* causes powdery mildew on a broad range of tree species. Infection of the rubber tree *Hevea brasiliensis* reduces yields of natural rubber. Several *E. quercicola* effectors have been identified and functionally characterized, and most suppress the plant immune system (Liu et al. 2025; Shan et al. 2025). Suppression of plant defenses is often critical for obligate biotrophs, as they form an intimate interface with a living host plant to complete their life cycle.

Strikingly, the *E. quercicola* effector Broad-spectrum Plant Immunity Elicitor 1 (EqBPIE1, previously EqCSEP04187) activates plant immunity (Li et al. 2022). Expression of EqBPIE1 in *H. brasiliensis* protoplasts or in the model plants *Nicotiana benthamiana* or *Arabidopsis thaliana* induced reactive oxygen species (ROS) accumulation and callose deposition (Li et al. 2022), two hallmarks of immune activation. Further, introduction of EqBPIE1 into the fungus *Colletotrichum gloeosporioides* reduced fungal virulence on *H. brasiliensis* (Li et al. 2022). In *E. quercicola,* EqBPIE1 is expressed specifically in the later stages of infection, and this temporal regulation appears to enable successful colonization by *E. quercicola* (Li et al. 2022).

In a recent study published in *Plant Physiology*, He et al. (2026) probed the molecular basis of the activation of plant immunity by EqBPIE1. First, they investigated the subcellular localization of the effector in the model plant *N. benthamiana.* The effector consists of an N-terminal signal peptide (SP), a chloroplast transit peptide (CTP) and a putative functional domain (FD) (Fig. 1). C-terminal GFP-tagged full length (FL) EqBPIE1, or a mature secreted (MS) version lacking the SP, were detected in the cytosol, chloroplasts, and nuclei. Given that EqBPIE1 was detected in multiple subcellular locations, the authors questioned whether the effector was simultaneously targeted to different compartments or sequentially trafficked between them. Subcellular fractionation of proteins from *H. brasiliensis* leaves identified both the MS and FD forms in chloroplasts, while only the FD form was found in the nucleus, and only the MS form in the cytosol (He et al. 2026). The results suggest a sequential trafficking pathway, where the MS version of EqBPIE1 is translocated into the host cell and targeted to the chloroplasts, where, following removal of the CTP, the FD moves into the nucleus (Fig. 1).

**Figure 1:**
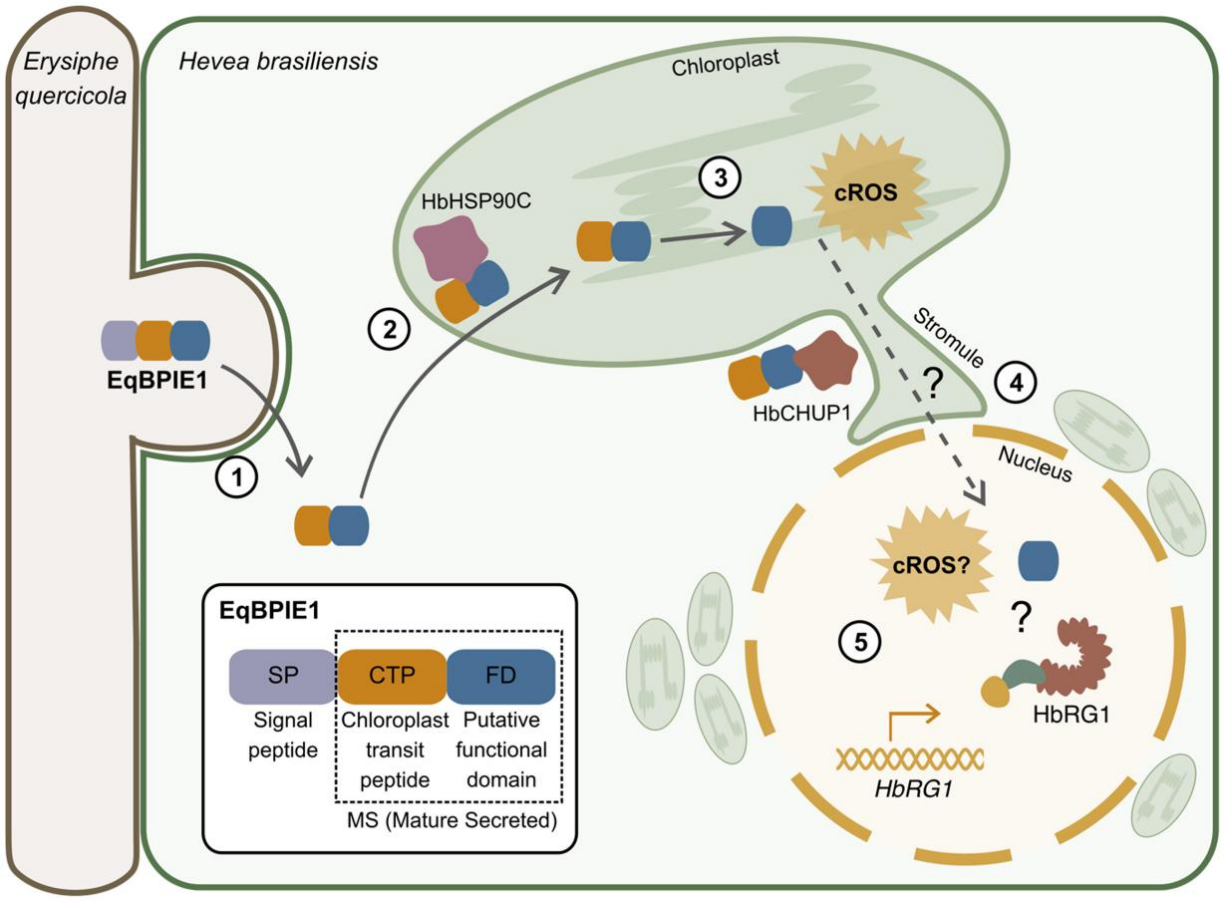
A model of sequential localization of EqBPIE1 to different subcellular compartments. Adapted from Figure 7 of He et al. (2026) (1) EqBPIE1 is translocated into *Hevea brasiliensis* cells. (2) The mature secreted form of EqBPIE1 is imported into the chloroplasts, assisted by the stromal chaperone HbHSP90C. (3) In chloroplasts, the chloroplast transit peptide is cleaved and EqBPIE1 activates ROS production. (4) EqBPIE1 triggers perinuclear chloroplast clustering and stromule formation, which may enable movement of ROS and/or the effector into the nucleus. (5) EqBPIE1-triggered changes in nuclear gene expression include the upregulation of the NLR immune receptor HbRG1, which may recognize EqBPIE1 or ROS to activate HR and resistance.

To identify host proteins associated with EqBPIE1, the authors leveraged co-immunoprecipitation coupled to mass spectrometry. One consistently enriched protein was HbHSP90C (Chloroplast Heat Shock Protein 90), a chloroplast-specific molecular chaperone. Import of preproteins to the chloroplast involves the TIC (Translocon on the Inner Chloroplast membrane) and TOC (Translocon on the Outer Chloroplast membrane) protein complexes. Stromal chaperones, such as HSP90C, associate with TIC and contribute to protein refolding and/or the ATP-dependent “pulling” of the translocated protein into the stroma (Inoue et al. 2013). After confirming the interaction between EqBPIE1 and HbHSP90C by co-immunoprecipitation, yeast two-hybrid (Y2H) and in vitro pulldown, He et al. (2026) demonstrated that treatment with an inhibitor of HbHSP90C decreased EqBPIE1 import into chloroplasts. Further, silencing *NbHSP90C* reduced EqBPIE1 accumulation in chloroplasts, but complementation with HvHSP90C largely restored EqBPIE1 chloroplast localization (He et al. 2026). Together, these data implicate HSP90C in the import of EqBPIE1 to chloroplasts (Fig. 1).

He et al. (2026) observed that expression of the MS version of EqBPIE1 in either *N. benthamiana* or *H. brasiliensis* induced perinuclear chloroplast clustering (PCC) and the formation of stromules, dynamic tubular structures which extend from plastids (He et al. 2026). Repositioning of chloroplasts around the nucleus and formation of stromules are well-known features of the plant immune response (Caplan et al. 2015). Multiple studies have indicated a pivotal role for chloroplast-derived reactive oxygen species (cROS) in plant immunity, both as potential retrograde signaling molecules and in mediating cell death through lipid peroxidation (reviewed by Littlejohn et al. 2021). Using the fluorescent probe DCFH-DA to detect ROS, He et al. found that expression of the MS version of EqBPIE1 induced chloroplast-derived ROS production ahead of PCC. Chloroplast localization of EqBPIE1 was shown to be necessary for both chloroplast-derived ROS production and PCC, as restricting the effector to the nucleus (by fusion to a nuclear localization signal) abolished both ROS production and PCC (He et al. 2026). The immunoprecipitation assay which identified HbHSP90C also pinpointed a second protein, HbCHUP1 (Chloroplast Unusual Positioning 1), which was confirmed by in vitro pulldown assays to be an interactor of EqBPIE1. CHUP1 is located on the outer chloroplast envelope and is involved in chloroplast repositioning (Oikawa et al. 2008). Silencing *NbCHUP1* did not affect accumulation of EqBPIE1 in the chloroplast, but significantly reduced PCC and nuclear accumulation of EqBPIE1. PCC and stromule formation appears to be a necessary prerequisite for the translocation of EqBPIE1 to the nucleus; treatment with an inhibitor of actin polymerization also compromised nuclear translocation of EqBPIE1 (He et al. 2026). Together, these data support a model whereby chloroplast-localized EqBPIE1 initiates cROS production, stromule formation, and PCC, which are required for the transfer of the effector into the nucleus (Fig. 1).

Next, the authors focused on the activity of nuclear-localized EqBPIE1. Spray treatment of *H. brasiliensis* with purified EqBPIE1 protein triggered significant transcriptional changes, including upregulation of defense-related genes. One such upregulated gene encoded a CC-NLR (Coiled-Coil–Nucleotide-binding, Leucine-rich Repeat) immune receptor designated *HbRG1* (Resistance Gene 1). Y2H and bimolecular fluorescence complementation experiments showed that EqBPIE1 interacts directly with HbRG1 (He et al. 2026). Notably, methionine-329 of HbRG1 was found to be oxidized in an EqBPIE1-dependent manner. It remains to be determined whether oxidative modification of HbRG1 primes the NLR for EqBPIE1 recognition, or whether the oxidative modification is itself the recognized event that leads to HbRG1 activation (Fig. 1).

Finally, the authors explored the potential of EqBPIE1 to activate defense responses in heterologous pathosystems. Pretreatment of *H. brasiliensis* leaves with purified EqBPIE1 protein increased resistance to 2 unrelated fungal pathogens. In addition, EqBPIE1 treatment reduced susceptibility of *N. benthamiana* and *Glycine max* to their oomycete pathogens *Phytopthora capsici* and *Phytopthora sojae,* respectively, and increased resistance of *A. thaliana* to the bacterial pathogen *Pseudomonas syringae* and the necrotrophic fungus *Botrytis cinerea* (He et al. 2026). Collectively, these data indicate that EqBPIE1 can activate plant defenses in an array of species.

By dissecting the mechanistic basis of EqBPIE1 activation of plant defenses, He et al. have highlighted the importance of temporal and spatial regulation of effectors in mediating successful infection. It is counterintuitive that an effector from an obligate biotroph activates plant immune signaling and the timing of effector expression appears critical for successful infection. The authors postulate that activation of host immunity at a late stage of the infection may confer an evolutionary advantage by ensuring the persistence of the host, and thus the pathogen. While He et al. have identified 2 proteins as interactors of EqBPIE1, it remains unclear how the effector triggers cROS following its import into the chloroplasts. Other effectors are known to target components of the photosynthetic electron transport chain to suppress cROS production (Rodríguez-Herva et al. 2012; Xu et al. 2019). The capacity of EqBPIE1 to promote pathogen resistance in a range of plant species suggests that the targeted components or processes are conserved. The potential biotechnological applications of this broad-spectrum defense-eliciting effector merit future investigation.

## Recent related articles in *Plant Physiology*

- (Lu et al. 2025) describe the immune-suppressing activity of an effector from the wheat stripe rust fungus *Puccinia striiformis* f. sp. *tritici* (Pst).
- (Gong et al. 2025) explored how an effector from the apple leaf spot fungus *Alternaria alternata* f. sp *mali* suppresses plant defenses.
- (Huai et al. 2026) demonstrated that an effector from the wheat powdery mildew fungus *Blumeria graminis* f. sp. *tritici* is recognized by the immune system of a non-host but contributes to fungal virulence on a wheat host.

## Data Availability

No new data included in this article.
